# Detection of SARS-CoV-2 and the L452R spike mutation using reverse transcription loop-mediated isothermal amplification plus bioluminescent assay in real-time (RT-LAMP-BART)

**DOI:** 10.1371/journal.pone.0265748

**Published:** 2022-03-21

**Authors:** Takahiro Iijima, Shinnosuke Ando, Dai Kanamori, Kazumichi Kuroda, Tsutomu Nomura, Laurence Tisi, Paul E. Kilgore, Neil Percy, Hikaru Kohase, Satoshi Hayakawa, Mitsuko Seki, Tomonori Hoshino

**Affiliations:** 1 Division of Pediatric Dentistry, Department of Human Development and Fostering, Meikai University School of Dentistry, Saitama, Japan; 2 Division of Dental Anesthesiology, Department of Diagnostic and Therapeutic Sciences, Meikai University School of Dentistry, Saitama, Japan; 3 Division of Gastroenterology and Hepatology, Department of Medicine, Nihon University School of Medicine, Tokyo, Japan; 4 Division of Otolaryngology, Department of Comprehensive Medical Sciences, Meikai University School of Dentistry, Saitama, Japan; 5 Erba Molecular, Erba Molecular–Lumora, Ely, United Kingdom; 6 Department of Pharmacy Practice, Eugene Applebaum College of Pharmacy & Health Sciences, Wayne State University, Detroit, MI, United States of America; 7 3M Company, St. Paul, MN, United States of America; 8 Division of Microbiology, Department of Pathology and Microbiology, Nihon University School of Medicine, Tokyo, Japan; Taif University, SAUDI ARABIA

## Abstract

The new coronavirus infection (COVID-19) caused by severe acute respiratory syndrome coronavirus 2 (SARS-CoV-2) can be fatal, and several variants of SARS-CoV-2 with mutations of the receptor-binding domain (RBD) have increased avidity for human cell receptors. A single missense mutation of U to G at nucleotide position 1355 (U1355G) in the spike (*S*) gene changes leucine to arginine (L452R) in the spike protein. This mutation has been observed in the India and California strains (B.1.617 and B.1.427/B.1.429, respectively). Control of COVID-19 requires rapid and reliable detection of SARS-CoV-2. Therefore, we established a reverse transcription loop-mediated isothermal amplification (RT-LAMP) assay plus a bioluminescent assay in real-time (BART) to detect SARS-CoV-2 and the L452R spike mutation. The specificity and sensitivity of the RT-LAMP-BART assay was evaluated using synthetic RNAs including target sequences and RNA-spiked clinical nasopharyngeal and saliva specimens as well as reference strains representing five viral and four bacterial pathogens. The novel RT-LAMP-BART assay to detect SARS-CoV-2 was highly specific compared to the conventional real-time RT-PCR. Within 25 min, the RT-LAMP-BART assay detected 80 copies of the target gene in a sample, whereas the conventional real-time RT-PCR method detected 5 copies per reaction within 130 min. Using RNA-spiked specimens, the sensitivity of the RT-LAMP-BART assay was slightly attenuated compared to purified RNA as a template. The results were identical to those of the conventional real-time RT-PCR method. Furthermore, using a peptide nucleic acid (PNA) probe, the RT-LAMP-BART method correctly identified the L452R spike mutation. This is the first report describes RT-LAMP-BART as a simple, inexpensive, rapid, and useful assay for detection of SARS-CoV-2, its variants of concern, and for screening of COVID-19.

## Introduction

Coronavirus disease-2019 (COVID-19), caused by the SARS-CoV-2, has spread globally [1]. Although more than a year has passed since the World Health Organization (WHO) declared a pandemic, COVID-19 has not yet been contained, and the emergence of mutant strains has resulted in further morbidity and mortality [2–7]. Several variants of SARS-CoV-2 with mutations of the receptor-binding domain (RBD) increase avidity to receptors on human cells [2, 3, 6, 8]. A missense mutation (U1355G in the *S* gene) of RBD, representing L452R, has been observed in the India and California strains (B.1.617 and B.1.427 / B.1.429, respectively) [9, 10].

Rapid and reliable detection of SARS-CoV-2 variants is required to control COVID-19. However, conventional RT-PCR and real-time RT-PCR methods are time-consuming (> 2 h), complicated, and require expensive apparatus. Moreover, these methods cannot easily distinguish single missense mutations, for which direct DNA sequencing is needed.

The RT-LAMP assay is a simple and rapid (performance time ≤ 1 h) alternative to the conventional RT-PCR and real-time RT-PCR methods [11]. RT-LAMP has been used previously to detect the coronaviruses that caused SARS and Middle East Respiratory Syndrome (MERS) [12, 13]. Generally, real-time monitoring using molecular assays including RT-PCR relies on fluorescence detection of the amplicon. These methods require complex and expensive apparatus, which limits the use of real-time PCR to well-equipped laboratories. Amplification in the RT-LAMP assay is monitored based on the turbidity induced by the precipitation of magnesium pyrophosphate with newly synthesized DNA. Using this mechanism, LAMP real-time turbidity monitoring is simpler than conventional real-time RT-PCR with fluorescence monitoring.

BART is a real-time, closed-tube luminescence reporter system that enables the progress of amplification to be monitored continuously using simple light measuring equipment [14]. Based on the bioluminescence output, it continuously reports the exponential increase in inorganic pyrophosphate produced during the isothermal amplification of a specific nucleic acid target. Because monitoring the BART reaction requires only a simple light detector, the combination of RT-LAMP plus BART has the potential to overcome the limitations of conventional RT-PCR or real-time RT-PCR methods, making it ideal for molecular diagnostic assays in laboratories with limited resources.

We established a novel RT-LAMP-BART method to detect SARS-CoV-2 (SARS-RT-LAMP-BART) and an RT-LAMP-BART method to identify genotypes with the L452R spike mutation (L452R-RT-LAMP-BART).

## Materials and methods

### Viral and bacterial strains

In total, 18 strains of five viral and four respiratory bacterial species were used to evaluate the RT-LAMP-BART (Table 1). The viral species were Influenza A [H1N1] (PR8 and Pdm2009) and respiratory syncytial virus (RSV; Long, subgroups A and B) (Prof. Tetsuo Nakayama, Kitasato University, Kanagawa, Japan); human coronavirus (OC43 and 229E) (American Type Culture Collection, Manassas, VA); SARS-CoV-2 (JPN/AI/1-004 and JPN/TY/WK-521) (National Institute of Infectious Diseases and Nihon University, Tokyo, Japan). The remaining human coronaviruses were provided by Vircell, Granada, Spain, including SARS-CoV-2 B.1.1.7 (hCoV-19/Spain/AN-HUSC_24581802/2020), B.1.351 (hCoV-19/Spain/GA-CHUVI-19118872/2020), P.1 (hCoV-19/Spain/GA-CHUVI-19250962/2021), B.1.617.2 (hCoV-19/Spain/GA-CHUVI-33984566/2021), and MERS-CoV (Betacoronavirus England 1). The respiratory bacterial species were *Neisseria meningitidis* (HY0001) (Hanyang University, Ansan, Korea), *Streptococcus pneumoniae* (6305), *Haemophilus influenzae* (Rd KW20), and *Escherichia coli* (DH5α).

**Table 1:** Reactivities and specificities of RT-LAMP-BART assays detecting *RdRp* gene and the L452R spike mutation of SARS-CoV-2.

Virus/Bacteria name	Results of two RT-LAMP-BART assays	
	*RdRp*	L452R
**SARS-CoV-2**		
JPN/AI/1-004 **^b^**	+ **^a^**	-
JPN/TY/WK-521 **^b^**	+	-
B.1.1.7 (ALPHA) hCoV-19/Spain/AN-HUSC_24581802/2020 **^c^**	+	-
B.1.351 (BETA) hCoV-19/Spain/GA-CHUVI-19118872/2020 **^c^**	+	-
P.1 (GAMMA) hCoV-19/Spain/GA-CHUVI-19250962/2021 **^c^**	+	-
B.1.617.2 (DELTA) hCoV-19/Spain/GA-CHUVI-33984566/2021 **^c^**	+	+
**MERS-CoV** Betacoronavirus England 1 **^c^**	-	-
**Human CoV** OC43 **^d^**	-	-
**Human CoV** 229E **^d^**	-	-
**Influenza A** (H1N1) PR8 **^e^**	-	-
**Influenza A** (H1N1) Pdm2009 **^e^**	-	-
**RSV** Long **^e^**	-	-
**RSV** subgroup A **^e^**	-	-
**RSV** subgroup B **^e^**	-	-
***Haemophilus influenzae*** Rd KW20	-	-
***Streptococcus pneumoniae*** 6305	-	-
***Neisseria meningitidis*** HY0001**^f^**	-	-
***Escherichia coli*** DH5α	-	-

^a^ +, positive; -, negative.

Source

^b^, National Institute of Infectious Diseases

^c^, Vircell

^d^, ATCC.

^e^, Kitasato University

^f^, Hanyang University.

### Preparation of genomic DNA/RNA

Genomic DNA from *S*. *pneumoniae*, *H*. *influenzae* and *E*. *coli* was extracted using a QIAamp DNA Mini kit (Qiagen, Valencia, CA) according to the manufacturer’s protocol. The genetic material of the five viral species and the *N*. *meningitidis* strain were transferred as purified RNA/DNA.

Genomic DNA was analyzed using a NanoDrop 1000/2000 instrument (Thermo Fisher Scientific Inc., Waltham, MA). The number of genome copies in the reaction mixture was calculated based on a molecular size of 2.04 Mbp for *S*. *pneumoniae* (R6; GenBank accession number, AE007317), 5.2 Mbp for *E*. *coli* (CFT073; GenBank accession number, AE014075.1), 1.83 Mbp for *H*. *influenzae* (RD KW20; GenBank accession number, NC000907), and 2.3 Mbp for *N*. *meningitidis* (MC58; GenBank accession number, AE002098).

The copy numbers of genomic RNA in SARS-CoV-2 were calculated using real-time RT-PCR [15]. Those of MERS-CoV, human coronavirus, influenza virus, and RSV were determined according to the manufacturer’s instructions. Each DNA/RNA sample was adjusted at least 10^4^ copies per microliter and used to evaluate assay specificity.

For the detection limit study, 1500 bases of synthetic SARS-CoV-2 RNAs including the target region of *RdRp*, *N*, or *S* gene (S1 Fig) were prepared, and the dilutions were amplified (5 × 10^4^, 5 × 10^3^, 500, 50, and 5 copies per reaction). Additionally, dilutions of 400, 300, 200, 100, 90, 80, 70, and 60 copies per reaction were used for the RT-LAMP-BART assay, and dilutions of 20, 10, 2, and 1 copy per reaction were used for real-time PCR. The results were compared between the RT-LAMP-BART and conventional real-time PCR methods [15]. Each reaction was performed in triplicate. According to the CLSI EP-17 guideline regarding the limit of detection (LoD) [16], assays were performed 20 times and the lowest level at which positive results were obtained in 95% of the assays (19/20 positive) was taken as the LoD. If less than 19/20 positive results were obtained for a particular copy number, LoD was estimated using the following equation adapted from a previous report [17], and its confidence interval was calculated using Clopper-Pearson analysis [18].


LoD=ln0.05/−(lnN/−C)

(N, fraction negatives of the test results; C, average expected copies subject to testing)

### LAMP primer design

The *RdRp* gene sequences of the following virus strains were compared [seven coronaviruses-SARS-CoV-2 (GenBank accession number, NC_045512.2), SARS-CoV (NC_004718.3), MERS-CoV (NC_019843.3), and four human coronaviruses (α-coronavirus; 229E and NL63, AF304460.1 and AY567487.2, respectively; β-coronavirus: OC43 and HKU1, AY391777.1 and NC_006577.2, respectively)] (S2 Fig). The Primer Explorer v. 5 software [19] was used and the *RdRp* gene (SARS-CoV-2 isolate Wuhan-Hu-1; GenBank accession number, NC_045512.2; S3 Fig) was targeted to design the LAMP primer set. The LAMP primer set included two outer primers (F3 and B3), two inner primers (FIP and BIP), and two loop primers (LF and LB) (Table 2). In addition, a primer set targeting L452R of the spike protein (*S* gene; hCoV-19/Japan/IC-0604/2021; GISAID accession ID, EPI_ISL_779690; S3 Fig) plus a peptide nucleic acid (PNA) probe for L452 (Table 2) were developed. The PNA probe was designed to be complementary to the wild-type sequence including a single nucleotide, T, at nucleotide position 1355 (T1355) of the *S* gene that is related to the leucine (L452) in the spike protein. This position was chosen to interfere with the annealing of the 3’ end of the FIP and the concomitant extension, also bioinformatics suggests no cross react with any other than SARS-CoV-2.

**Table 2:** LAMP primer sets and a probe used in this study.

	Sequence 5’-3’
**RdRp-RT-LAMP-BART primer**	
**RdRp_F3**	AAA ACG TAA TGT CAT CCC TAC
**RdRp_B3**	AGT TTT TAA CAT GTT GTG CCA
**RdRp_FIP**	TAC AGA TAG AGA CAC CAG CTA CGC TCA AAT GAA TCT TAA GTA TGC C
**RdRp_BIP**	ACT ATG ACC AAT AGA CAG TTT CAT CTT CCA ATT ACT ACA GTA GCT CCT C
**RdRp_LF**	GCG AGC TCT ATT CTT TGC A
**RdRp_LB**	GAA ATC AAT AGC CGC CAC
**L452R-RT-LAMP-BART primer and PNA probe**	
**L452R_F3**	GCA AAC TGG AAA GAT TGC TGA T
**L452R_B3**	TTG GAA ACC ATA TGA TTG TAA AGG A
**L452R_FIP**	CGG TAA TTA TAA TTA CCA CCA ACC TAG ATG ATT TTA CAG GCT GCG T
**L452R_BIP**	GTT TAG GAA GTC TAA TCT CAA ACC TAA CAC CAT TAC AAG GTG TGC TA
**L452R_LF**	TCA AGA TTG TTA GAA TTC CAA GCT AT
**L452R_LB**	AGA GAG ATA TTT CAA CTG AAA TCT ATC AG
**L452R_PNA probe**	CCT AAA CAA TCT ATA CA ^a^ G G

^a^, the PNA clamping probe was designed to be complementary to the wild-type sequence (U1355 of *S* gene) and positioned to interfere the RT-LAMP reaction.

Red text, L452R (T1355G).

### RT-LAMP-BART assay

Lyophilized BART Master™ Reagent (Erba Mannheim, Ely, UK) was used as a diluent to produce 1.6 μM FIP and BIP, 0.4 μM F3 and B3, 0.8 μM LF and LB, and 2 × 125 mM bicine acetate buffer with 2 mM MgCl_2_. For the L452R-RT-LAMP-BART assay, 100 nM of PNA was added. The final reagent mix was adjusted with medical grade water (MGW) to obtain a 15-μL volume. To this volume, 5 μL of template DNA/RNA was then added (20 μL total volume). Reaction mixtures were covered with mineral oil to prevent evaporation.

The reaction tube was incubated at 60°C for 60 min and the light output from each tube as a function of time using the real-time LAMP-BART apparatus (PCRuN, Biogal Galed Lab, Kibbutz Galed, Israel or 3M™ Molecular Detection Instrument, MDS100, 3M, MN) was monitored.

After an initial decrease in light during the first few minutes, the samples were considered positive if the light signal increased markedly, followed by a decrease in the light output [14]. The peak light output in the assay was identified in accordance with each manufacturer’s protocol. Amplified products were also observed with the naked eye.

### Analysis of RT-LAMP-BART products

Amplified LAMP products were sequenced at the Akita Prefectural University Biotechnology Center using a BigDye Terminator v. 3.1 cycle sequencing kit (Applied Biosystems, Foster City, CA) on a 3130xL genetic analyzer (Applied Biosystems). The following F2 primers for SARS-RT-LAMP and L452R-RT-LAMP were used to sequence the target regions: RdRp-F2, 5′-CTC AAA TGA ATC TTA AGT ATG CC-3′; and L452R-F2, 5′-AGA TGA TTT TAC AGG CTG CGT-′.

### Real-time RT-PCR

Real-time RT-PCR (TaqMan probe method) was performed using a One Step Prime Script RT-PCR kit (TaKaRa Bio, Shiga, Japan) with primers and a probe (N2 set [15]) (S1 Table). The cycling conditions of the real-time RT-PCR were as follows: reverse-transcription at 50°C for 30 min; denaturation at 95°C for 15 min; 45 cycles of amplification (at 95°C for 15 s, and at 60°C for 60 s); and then 40°C for 30 s (cooling) using the LightCycler480 system (Roche Diagnostics, Basel, Switzerland).

### RNA-spiked specimens

The sensitivity of the LAMP assay was evaluated using RNA-spiked clinical samples, including nasopharyngeal swab (NPS) and saliva (SAL) specimens, obtained from five healthy volunteers.

The NPS specimens were collected using FLOQSwab^TM^534CS01-E and Universal Transport Medium (UTM^®^; Copan, Brescia, Italy), and stored at –2°C. SAL specimens were collected in sterilized tubes and frozen at –20°C. NPS and SAL samples were subjected to Loopamp™ Virus RNA extraction reagent (Eiken Chemical Co., Tokyo, Japan) according to the manufacturer’s instructions. Synthetic SARS-CoV-2 RNAs were spiked into the specimens and used to evaluate LoDs.

### Ethics statement

We collected NPS and SAL specimens from five healthy volunteers at the Meikai University School of Dentistry. The study protocol was reviewed and approved by the Institutional Review Board of the Meikai University School of Dentistry (IRB # A2001). Written informed consent was obtained from the five healthy volunteers.

## Results

### Assay reactivity and specificity

**Fig 1:**
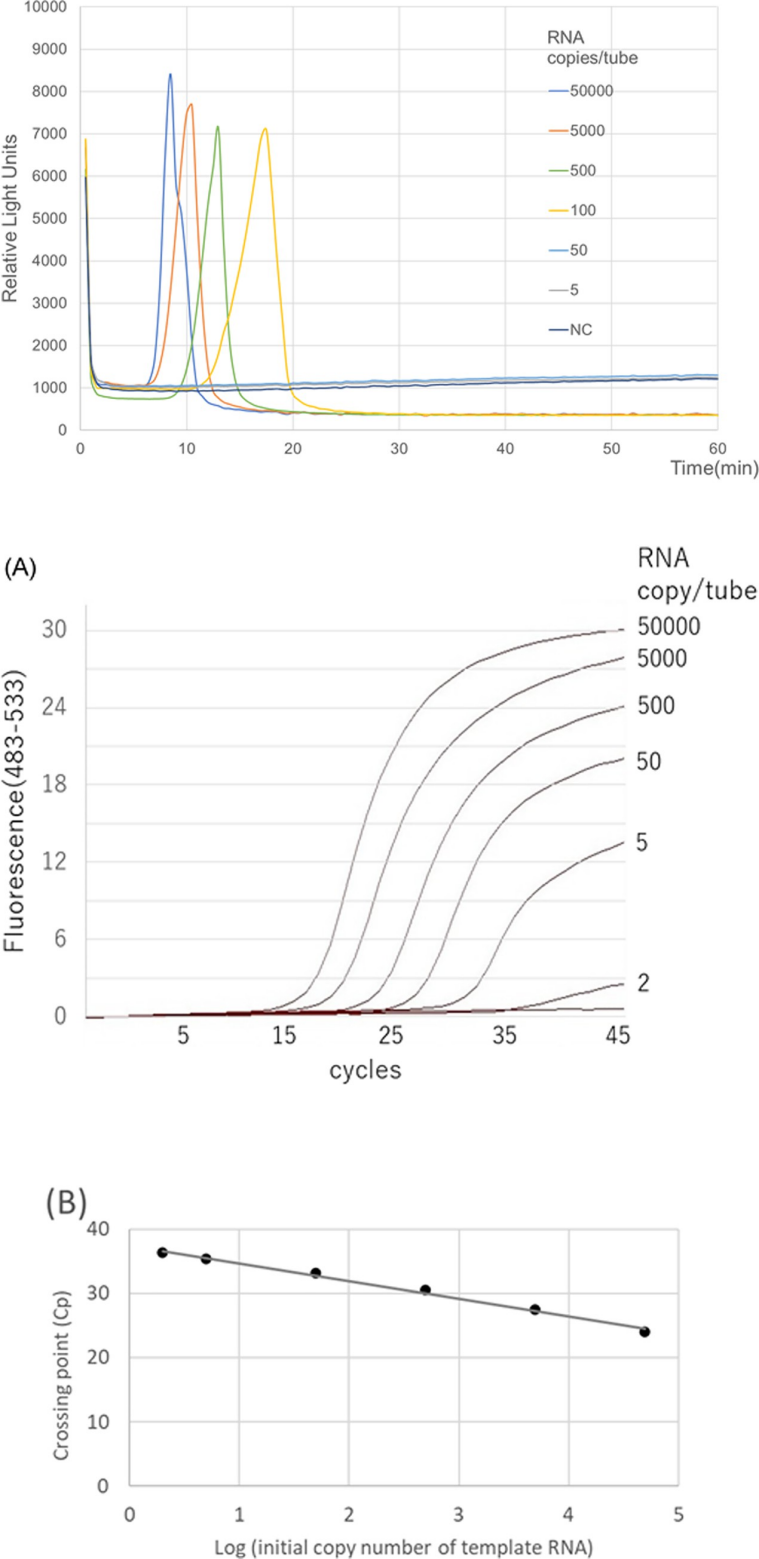
**A.** SARS-RT-LAMP-BART assay data derived via real-time monitoring the lights on the tube. Serial 10-fold-diluted synthetic SARS-CoV-2 RNA including the target region of *RdRp* and *N* genes (5×10^4^, 5×10^3^, 5×10^2^, 10^2^, 5×10, and 5 RNA copies) were assayed. The real-time monitoring was performed using Real-time LAMP-BART apparatus (PCRuN, Biogal Galed Lab, Israel). The assay was identified the light output peak in accordance with the manufacturer’s protocol. **B.** The Real-time RT-PCR (TaqMan probe method) data performed using a commercially available kit, One Step Prime Script RT-PCR kit (Takara Bio, Shiga, Japan), and LightCycler480 (Roche Diagnostics, Basel, Switzerland). Its reaction time was 130 min. (A) Serial 10-fold-diluted synthetic SARS-CoV-2 RNA including the target region of *RdRp* and *N* genes (5×10^4^, 5×10^3^, 5×10^2^, 5×10, and 5 RNA copies) plus 2 copies were assayed. (B) The relation between the Crossing point (Cp) of each sample and the log of the amount of initial template RNA.

The amplification of the RT-LAMP-BART assay was confirmed by the observation of turbidity in the reaction tube and by real-time monitoring of light output in the tube (Fig 1). Among the respiratory pathogens, the assay detected only SARS-CoV-2 (Table 1). No false-positive reaction occurred. The product was subjected to direct sequencing, and the sequences were compared with those of the targeted region (bases 1667–1716) of the *RdRp* gene (F1 to B1c, S3 Fig). The obtained sequences were as expected (S4 Fig), indicating that RT-LAMP-BART assay is highly specific for SARS-CoV-2.

Additionally, the *RdRp* gene sequences of eleven SARS-CoV-2 variants were compared with the wild-type, and the SARS-RT-LAMP-BART assay was expected to detect all these variants in silico (S2 Fig). The variants included B.1.1.7 (Alpha), GISAID accession ID, EPI_ISL_601443; B.1.351 (Beta), EPI_ISL_660190; P.1 (Gamma), EPI_ISL_906081; B.1.617.2 (Delta), EPI_ISL_1419152; B.1.427 (Epsilon), EPI_ISL_8750789; P.2 (Zeta), EPI_ISL_8005633; B.1.525 (Eta), EPI_ISL_1083809; B.1.526 (Iota), EPI_ISL_8158591; B.1.621 (Mu), EPI_ISL_8259884; B.1.529.1 (Omicron BA.1), EPI_ISL_6841980; and B.1.529.2 (Omicron BA.2), EPI_ISL_9097416.

### Limit of Detection

The SARS-RT-LAMP-BART assay detected 80 gene copies in a sample within 25 min of initiating sample testing, whereas the conventional real-time RT-PCR method detected 5 copies per reaction within 130 min (Table 3, Fig 1). Although the SARS-RT-LAMP-BART assay was less sensitive than conventional real-time RT-PCR, it produced results more rapidly. Moreover, the LAMP products could be measured based on light output in the reaction tube in real-time (Fig 1).

**Table 3:** Detection limits and reaction time of the real-time RT-PCR and RT-LAMP-BART assays used to detect synthetic SARS-CoV-2 RNA including the target region of *RdRp* and *N* genes and the synthetic RNA-spiked nasopharyngeal and saliva specimens.

	Limit of Detection (LoD)			
	Real-time RT-PCR (Reaction time, 130 min)		RT-LAMP-BART (Reaction time, 25 min)	
Purified RNA	5^a^ (19/20) ^b^	5^c^ (2.2–10.7)	80 ^a^ (19/20) ^b^	80 ^c^ (35.9–172.3)
**RNA spiked specimens**				
**Nasopharyngeal swab^d^**	10 (20/20)	9.3 (5.2–18.1)	200 (19/20)	200 (89.8–430.6)
	5 (16/20)			
**Saliva^d^**	50 (20/20)	43.2 (24.5–84.2)	300 (19/20)	300 (134.7–645.9)
	20 (15/20)			

^a^ amount of RNA copies per reaction

^b^ number of positive per 20 times trial (cutoff, Cp > 36).

^c^ estimated LOD (95% confidence interval)

^d^ samples prepared via Loopamp^TM^ Virus RNA extraction reagent (Eiken Chemical Co. LTD).

The estimated real-time RT-PCR LoDs for 95% positive call rates with 95% confidence intervals (95% CIs) were: 5 RNA copies per reaction (95% CI, 2.2–10.7) for purified RNA as a template, 9.3 RNA copies per reaction (95% CI, 5.2–18.1) for RNA-spiked NPS samples, and 43.2 RNA copies per reaction (95% CI, 24.5–84.2) for RNA-spiked SAL samples (Table 3). The estimated SARS-RT-LAMP-BART LoDs for 95% positive results were 80 RNA copies per reaction (95% CI, 35.9–172.3) for purified RNA as a template, 200 RNA copies per reaction for RNA-spiked NPS samples (95% CI, 89.8–430.6), and 300 RNA copies per reaction for RNA-spiked SAL samples (95% CI, 134.7–645.9). The RT-LAMP-BART assay and the conventional real-time RT-PCR method detected each target gene with slightly attenuated sensitivity compared to purified RNA as the template. Interestingly, it seems three LoDs of real-time RT-PCR were split into two independent groups (Purified RNA & NPS versus SAL). However, three LoDs of SARS-RT-LAMP-BART were not separate from each other. The attenuation range in SARS-RT-LAMP-BART might be relatively minor compared with real-time PCR.

Here, the SAL/NPS specimens were prepared via the Loopamp™ Virus RNA Extraction Reagent (Eiken Chemical Co. Ltd.), and then synthetic SARS-CoV-2 RNAs were spiked into the specimens and used for evaluation. This sample preparation way is rapid (1 min), and centrifugation is not required, but biological substances were not completely removed, especially from saliva.

### L452R-RT-LAMP-BART assay

**Fig 2:**
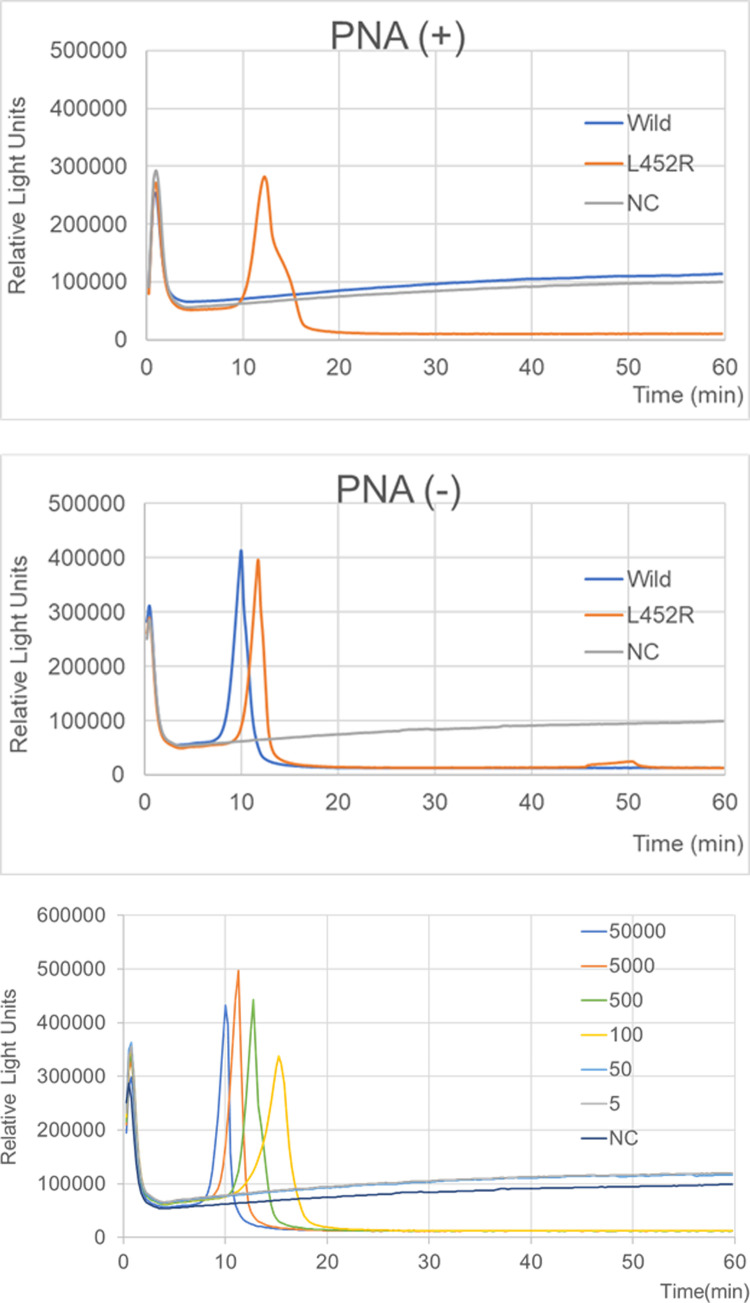
**A.** L452R-RT-LAMP-BART assay data with PNA and without PNA derived via real-time monitoring the lights on the tube using 3M™ Molecular Detection Instrument (MDS100, 3M, USA). Assayed samples were synthetic *S* gene RNA of SARS-CoV-2 with L452R (T1355G) (positive control, 5×10^4^ RNA copies), SARS-CoV-2 RNA (wild-type, JPN/AI/1-004, 5×10^6^ RNA copies), and DW. **B.** L452R-RT-LAMP-BART assay data derived via real-time monitoring the lights on the tube. Serial 10-fold-diluted samples (synthetic *S* gene RNA of SARS-CoV-2 with L452R (T1355G); 5×10^4^, 5×10^3^, 5×10^2^, 10^2^, 5×10, and 5 RNA copies) were assayed. The real-time monitoring was performed using Real-time LAMP-BART apparatus (MDS100, 3M, USA). The assay was identified the light output peak in accordance with the manufacturer’s protocol.

Of the six SARS-CoV-2 RNAs, including those from two wild-type viruses and four variants (B.1.1.7, B.1.351, P1, and B.1.617.2), only B.1.617.2 RNA was detected by the L452R-RT-LAMP-BART assay (Table 1). Using the PNA probe, we discriminated the U1355G mutation in the *S* gene from the other variants and wild-type SARS-CoV-2. We demonstrated that 5×10^4^ copies of the L452R sequence could be detected around 10 minutes whereas 5×10^6^ copies of wild-type remained undetected after 30 minutes (Fig 2A). Within 25 min, we detected down to 10^2^ RNA copies per reaction of the L452R sequence using the L452R-RT-LAMP-BART assay (Fig 2B). The product was subjected to direct sequencing. The sequences were compared to those of the targeted region (bases 1331–1389) of the *S* gene (F1 to B1c, S3 Fig). The 3′-end of the F1 region matched the L452R (T1355G) sequence (S4 Fig), and the other sequences were identical to those expected (S3 Fig).

## Discussion

We established a novel RT-LAMP-BART assay for SARS-CoV-2. Our SARS-RT-LAMP-BART assay is specific and sensitive for rapid identification of SARS-CoV-2. The *RdRp* gene of SARS-CoV-2 was correctly identified in 18 reference strains, including SARS-CoV-2 (wild-type and four variants), MERS-CoV, human coronavirus, RSV, and four respiratory bacterial pathogens.

Because COVID-19 has spread worldwide, rapid and accurate point-of-care testing/laboratory assessment will continue to be a high priority globally. For screening of COVID-19, the test’s speed and frequency are more important than its sensitivity [20]. In patients with COVID-19, the SARS-CoV-2 viral load rapidly increases before and just after symptom onset and decreases significantly thereafter. If the testing frequency is low, the virus cannot be detected, irrespective of the assay sensitivity.

In this study, the SARS-RT-LAMP-BART assay had lower sensitivity than the real-time RT-PCR in terms of the number of RNA copies detected. An alternative LAMP primer set targeting other regions could be included in the reaction mixture to increase the sensitivity, but that would make the reaction more complex and cause dimer accumulation, which may lead to false positive results.

RT-LAMP is simple and rapid (< 1 h) and has been used to detect the viruses causing SARS and MERS [12, 13]. The principle of BART is continuous monitoring of the enzymatic conversion of byproducts (PPi) of nucleic acid amplification into ATP by means of the bioluminescence generated by firefly luciferase [14]. The assay shows a unique kinetic signature (a light output peak) whose timing is dependent on the concentration of the target nucleic acid, on which is based the determination of positivity/negativity. Within 25 min, the RT-LAMP-BART assay detected up to 80 gene copies per reaction, whereas the conventional real-time RT-PCR method detected 5 copies per reaction in 130 min. In BART, the time for peak determination is short, thus obtaining results is easy and quick, and light detection can be performed at a low cost. In fact, the hardware required for the LAMP-BART assay consists simply of an isothermal heating block and a photodiode-based light detection system. This makes LAMP-BART hardware the simplest available solution for real-time detection of amplification. This simplicity offers significant cost reduction in the readers since no light source is needed for irradiating the samples, no expensive filter sets are required and no thermocycling electronics are required. Further, the method works with standard off-the-shelf consumables and the cost of the BART reporter system is equivalent to the cost of fluorescent reporter systems. There is also no need for a fluorescence/quenching probe, which is relatively expensive. The RT-LAMP-BART assay shows potential for rapid and frequent testing for COVID-19.

In this study, RT-LAMP-BART reactions were inhibited by biological substances in RNA-spiked samples, as in the conventional real-time RT-PCR method. In our previous studies, such interference from biological substances was not observed in LAMP reactions [21–23]. BART also has robustness to biological substances. Gandelman *et al*. reported that LAMP-BART showed robust behavior, reliable detection using urine specimens, and was not susceptible to inhibition by rapid DNA preparation [14]. However, in this study, RT-LAMP sensitivity was slightly attenuated by biological substances. We think that reverse transcription (RT) might have influenced these results, and may be key to improving the RT-LAMP reaction.

The manufacturers recommend using semi-alkaline protease or 1/10 dilution with saline/PBS for saliva-sample preparation, although the amount of RNA was also attenuated in the sample. The semi-alkaline proteinase is a sputum homogenizer, typically used for sample preparation in tuberculosis testing. The use of this enzyme for saliva-sample preparation was also recommended for RT-LAMP point-of-care testing [24]. The semi-alkaline protease could improve detection from saliva samples.

The B.1.617 variant is highly infectious and has caused outbreaks globally [25, 26]. In the United Kingdom, two doses of the BNT162b2 vaccine had an effectiveness of 87.9% against symptomatic disease caused by the B.1.617.2 COVID-19 variant [27]. A study in Scotland concluded that the vaccine was 79% effective against the variant [28]. Researchers in Canada measured its effectiveness at 87% [29]. Since June 6, 2021, a marked decline in the effectiveness of the vaccine in preventing infection (64%) and symptomatic illness (64%) has been observed simultaneously with the spread of the B.1.617.2 variant in Israel [30].

The B.1.617 variant has a typical point mutation in the RBD protein (L452R) that increases infectivity and resistance to current vaccines. The developed method is able to detect this mutation.

In principle, variant detection, especially indel or missense mutations, is not easy using other nucleic acid-based methods. Conventional RT-PCR and real-time RT-PCR methods also cannot easily identify a single missense mutation, for which direct sequencing is essential [22].

Nielsen *et al*. created the synthetic DNA analog PNA in 1991 [31]. The deoxyribose phosphate backbone is replaced by a pseudo-peptide polymer, to which the nucleobases are linked. PNA has no direct interaction with DNA polymerase, and hybridization of PNA to DNA/RNA occurs with remarkably high affinity and specificity. Thus, PNA can terminate the elongation of oligonucleotide primers by binding to the template or competing with the primers. Therefore, we used PNA as a molecular hybridization probe to inhibit amplification of the specific target.

LAMP plus PNA probe detection methods have been developed [32, 33]. In this study, the L452R-RT-LAMP-BART method using a PNA probe correctly identified the L452R spike mutation within 30 min, with an LoD of ~100 copies per reaction. To our knowledge, this is the first report of an RT-LAMP-BART assay using PNA to detect a mutation.

The PNA probe and RT-LAMP-BART method can also be applied to detect other SARS-CoV-2 variants. BART cannot be multiplexed conventionally, but its ease, low cost, and the simple image analysis required allow multiple reactions to be run simultaneously [14]. The method will be useful for variant detection after confirming SARS-CoV-2 infection, for which it would be helpful if the reaction time could be reduced to 25–30 min.

This study had some limitations. The sensitivity of SARS-RT-LAMP-BART was not as high as that of real-time RT-PCR. If COVID-19 is suspected clinically but negative results are obtained, more sensitive and available detection methods should be considered. Based on the alignment of the *RdRp* genes of seven coronaviruses, we also expect the SARS-RT-LAMP-BART assay to detect SARS-CoV (S2 Fig). Both species are serious respiratory pathogens. Further work using clinical specimens from patients is required.

## Conclusions

This is the first report of a SARS-CoV-2 detection assay using the RT-LAMP-BART method, which will facilitate COVID-19 screening and the identification of infection with SARS-CoV-2 variants of concern or interest. In contrast with the use of DNA templates, the sensitivity of RT-LAMP can be diminished by biological substances. This should be considered when using a simple RNA extraction method.

## Supporting information

S1 Fig A), synthetic SARS-CoV-2 RNA including the target region of *RdRp* and *N* genes; B), synthetic SARS-CoV-2 RNA including the target region of *S* gene.
(PDF)

S2 Fig Nucleotide sequences of SARS-CoV-2 *RdRp* gene used for SARS-RT-LAMP primers in this study (·, consensus sequence between SARS-CoV-2 isolate Wuhan-Hu-1). (PDF)

S3 Fig A), Nucleotide sequence of the SARS-CoV-2 *RdRp* gene used to design the SARS-RT-LAMP-BART primers; B), Nucleotide sequence of the SARS-CoV-2 *S* gene used to design the L452R-RT-LAMP-BART primers. The sequences used for the RT-LAMP primers are indicated by arrows.
(PDF)

S4 Fig Sequence data of amplified products of (A) SARS-RT-LAMP-BART and (B) L452R-RT-LAMP-BART assays.
(PDF)

S1 Table Primer set for real-time RT-PCR [15]. (PDF)
