# Supplementary material for: Detection of SARS-CoV-2 and the L452R spike mutation using reverse transcription loop-mediated isothermal amplification plus bioluminescent assay in real-time (RT-LAMP-BART)
Source: PLoS One. 2022 Mar 21;17(3):e0265748. doi: 10.1371/journal.pone.0265748 (PMC8936440; doi:10.1371/journal.pone.0265748)
Supplement: S1 Fig — A), synthetic SARS-CoV-2 RNA including the target region of RdRp and N genes; B), synthetic SARS-CoV-2 RNA including the target region of S gene. (PDF) [file pone.0265748.s001.pdf]

Fig. S1. A), synthetic SARS-CoV-2 RNA including the target region of *RdRp* and *N* genes; B), synthetic SARS-CoV-2 RNA including the target region of *S* gene.

A)

gggAAUGAGUUAUGAGGAUCAAGAUGCACUUUUCGCAUAUACAAAACGUAAUGUCA
UCCCUACUAUAACUCAAAUGAAUCUUAAGUAUGCCAUUAGUGCAAAGAAUAGAGC
UCGCACCGUAGCUGGUGUCUCUAUCUGUAGUACUAUGACCAAUAGACAGUUUCAU
CAAAAAUUAUUGAAAUCAAUAGCCGCCACUAGAGGAGCUACUGUAGUAAUUGGAA
CAAGCAAAUUCUAUGGUGGUUGGCACAACAUGUUAAAAACUGUUUAUAGUGAUGU
AGAAAACCCUCACCUUAUGGGUUGGGAUUAUCCUAAAUGUGAUAGAGCCAUGCCU
AACAUGCUUAGAAUUAUGGCCUCACUUGUUCUUGCUCGCAAACAUACAACGUGUU
GUAGCUUGUCACACCGUUUCUAUAGAUUAGCUAAUGAGUGUGCUCAAGUAUUGA
GUGAAAUGGUCAUGUGUGGCGGUUCACUAUAUGUUAAACCAGGUGGAACCUCAU
CAGGAGAUGCCACAAUUUUGCUCUCAACAUACAAUGCUAGUUAAACAGGGUGAUG
AUUAUGUGUACCUUCCUUACCCAGAUCCAUCAAGAAUCCUAGGGGCCGGCUGUU
UUGUAGAUGAUAUCGUAAAAACAGAUGGUACACUUAUGAUUGAACGGUUCGUGUC
UUUAGCUAUAGAUGCUUACCCACUUACUAAACAUCCUAAUCAGGAGUAUGCUGAU
GUCUUUCAUUUGUACUUACAAUACAUAAGAAAGCUACAUGAUGAGUUAACAGGAC
ACAUGUUAGACAUGUAUUCUGUUAUGCUUACUAAUGAUAACACUACGUUUGGUGG
ACCCUCAGAUUCAACUGGCAGUAACCAGAAUGGAGAACGCAGUGGGGCGCGAUC
AAAACAACGUCGGCCCCAAGGUUUACCCAAUAAUACUGCGUCUUGGUUCACCGCU
CUCACUCAACAUGGCAAGGAAGACCUUAAAUUCCCUCGAGGACAAGGCGUUCCAA
UUAACACCAAUAGCAGUCCAGAUGACCAAAUUGGCUACUACCGAAGAGCUACCAG
ACGAAUUCGUGGUGGUGACGGUAAAAUGAAAGAUCUCAGUCCAAGAUGGUAUUUC
UACUACCUAGGAACUCACAAGCUUUCGGCAGACGUGGUCCAGAACAAACCCAAGG
AAAUUUUGGGGACCAGGAACUAAUCAGACAAGGAACUGAUUACUGGCAUGGAAGU
CACACCUUCGGGAACGUGGUUGACCUACACAGGUGCAAACAUUGGCCGCAAAUU
GCACAAUUUGCCCCCAGCGCUUCAGCGUUCUUCGGAAUGUCGCGCAUCAUCAAA
UUGGAUGACAAAGAUCCAAAUUUCAAAGAUCAAGUCAUUUUGCUGAAUAAGCAUA
UUGACGCAUACAAAACAUUCCCACCAACAGAGCCUAAAAAGGACAAAAAGAAGAAG
GCUGAUGAAAAACUGUGACUCUUCUUCCUGCUGCAGAUUUGGCUCAAGCCUUAC
CGCAGAGACAGAAGAAACAGCA

B)

gggAGAAACAAAGUGUACGUUGAAAUCCUUCACUGUAGAAAAAGGAAUCUAUCAA
ACUUCUAACUUUAGAGUCCAACCAACAGAAUCUAUUGUUAGAUUUCCUAAUAUU
ACAAACUUGUGCCCUUUUGGUGAAGUUUUUAACGCCACCAGAUUUGCAUCUGU
UUAUGCUUGGAACAGGAAGAGAAUCAGCAACUGUGUUGCUGAUUAUUCUGUCC
UAUAUAAUUCCGCAUCAUUUUCCACUUUUAAGUGUUAUGGAGUGUCUCCUACUA
AAUUAAAUGAUCUCUGCUUUACUAAUGUCUAUGCAGAUUCAUUUGUAAUUAGAG
GUGAUGAAGUCAGACAAAUCGCUCCAGGGCAAACUGGAAAGAUUGCUGAUUAUA
AUUAUAAAUUACCAGAUGAUUUUACAGGCUGCGUUAUAGCUUGGAAUUCUAACA
AUCUUGAUUCUAAGGUUGGUGGUAAUUAUAAUUACC**G***GUAUAGAUUGUUUAGG
AAGUCUAAUCUCAAACCUUUUGAGAGAGAUAUUUCAACUGAAAUCUAUCAGGCC
GGUAGCACACCUUGUAAUGGUGUUCAAGGUUUUAAUUGUUACUUUCCUUUACA
AUCAUAUGGUUUCCAACCCACUAAUGGUGUUGGUUACCAACCAUACAGAGUAGU
AGUACUUUCUUUUGAACUUCUACAUGCACCAGCAACUGUUUGUGGACCUAAAAA
GUCUACUAAUUUGGUUAAAAACAAAUGUGUCAAUUUCAACUUCAAUGGUUUAAC
AGGCACAGGUGUUCUUACUGAGUCUAACAAAAAGUUUCUGCCUUUCCAACAAUU
UGGCAGAGACAUUGCUGACACUACUGAUGCUGUCCGUGAUCCACAGACACUUG
AGAUUCUUGACAUUACACCAUGUUCUUUUGGUGGUGUCAGUGUUAUAACACCA
GGAACAAAUACUUCUAACCAGGUUGCUGUUCUUUAUCAGGAUGUUAACUGCACA
GAAGUCCCUGUUGCUAUUCAUGCAGAUCAACUUACUCCUACUUGGCGUGUUUA
UUCUACAGGUUCUAAUGUUUUUCAAACACGUGCAGGCUGUUUAAUAGGGGCUG
AACAUGUCAACAACUCAUAUGAGUGUGACAUACCCAUUGGUGCAGGUAUAUGCG
CUAGUUAUCAGACUCAGACUAAUUCUCCUCGGCGGGCACGUAGUGUAGCUAGU
CAAUCCAUCAUUGCCUACACUAUGUCACUUGGUGCAGAAAAUUCAGUUGCUUAC
UCUAAUAACUCUAUUGCCAUACCCACAAAUUUUACUAUUAGUGUUACCACAGAA
AUUCUACCAGUGUCUAUGACCAAGACAUCAGUAGAUUGUACAAUGUACAUUUGU
GGUGAUUCAACUGAAUGCAGCAAUCUUUUGUUGCAAUAUGGCAGUUUUUGUAC
ACAAUUAAACCGUGCUUUAACUGGAAUAGCUGUUGAACAAGACAAAAACACCCA
AGAAGUUUUUGCACAAGUCAAACAAAUUUACAAAACACCACCAAUUAAAGAUUU

*, L452R (U1355G)
