# Supplementary material for: Detection of SARS-CoV-2 and the L452R spike mutation using reverse transcription loop-mediated isothermal amplification plus bioluminescent assay in real-time (RT-LAMP-BART)
Source: PLoS One. 2022 Mar 21;17(3):e0265748. doi: 10.1371/journal.pone.0265748 (PMC8936440; doi:10.1371/journal.pone.0265748)

**Fig. S2. Nucleotide sequences of SARS-CoV-2 *RdRp* gene used for SARS-RT-LAMP primers in this study** (·, consensus sequence between SARS-CoV-2 isolate Wuhan-Hu-1).

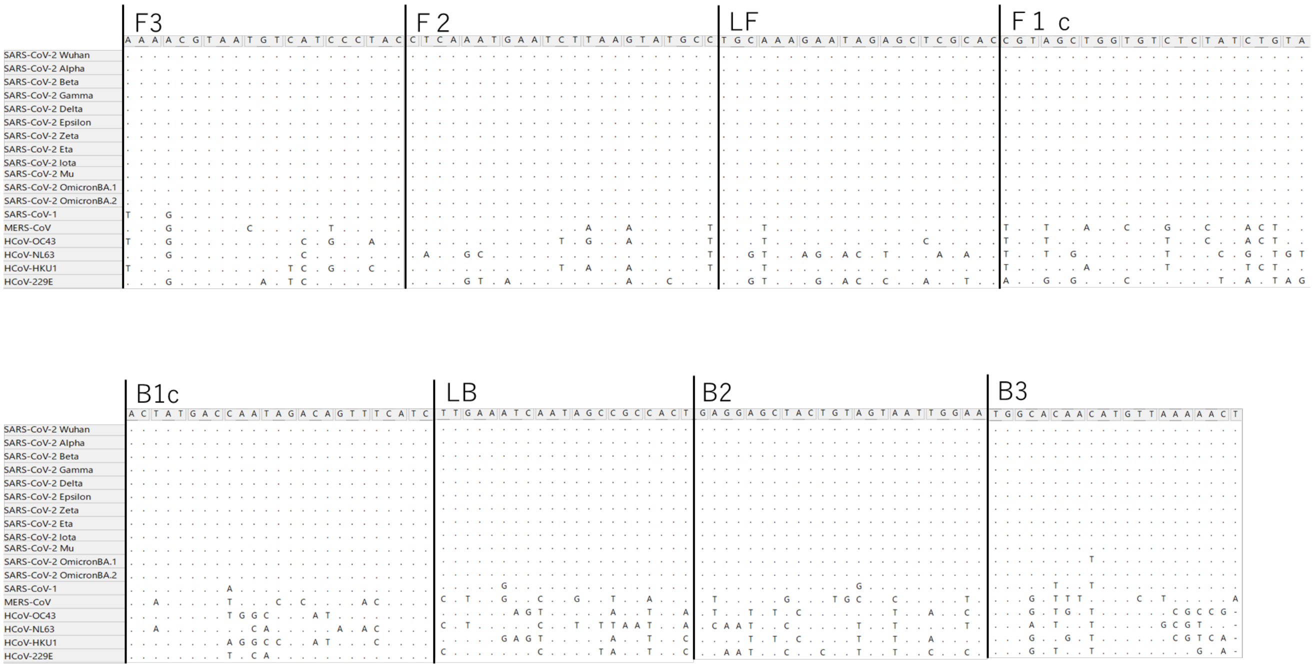

Supplement: S2 Fig — (PDF) [file pone.0265748.s002.pdf]
