# Supplementary material for: Detection of SARS-CoV-2 and the L452R spike mutation using reverse transcription loop-mediated isothermal amplification plus bioluminescent assay in real-time (RT-LAMP-BART)
Source: PLoS One. 2022 Mar 21;17(3):e0265748. doi: 10.1371/journal.pone.0265748 (PMC8936440; doi:10.1371/journal.pone.0265748)
Supplement: S3 Fig — A), Nucleotide sequence of the SARS-CoV-2 RdRp gene used to design the SARS-RT-LAMP-BART primers; B), Nucleotide sequence of the SARS-CoV-2 S gene used to design the L452R-RT-LAMP-BART primers. The sequences used for the RT-LAMP primers are indicated by arrows. (PDF) [file pone.0265748.s003.pdf]

Fig. S3. A), Nucleotide sequence of the SARS-CoV-2 *RdRp* gene used to design the SARS-RT-LAMP-BART primers; B), Nucleotide sequence of the SARS-CoV-2 *S* gene used to design the L452R-RT-LAMP-BART primers. The sequences used for the RT-LAMP primers are indicated by arrows.

A)

```
NO          1591
Sequence    CAAAACGTAA TGTCATCCCT ACTATAACTC AAATGAATCT TAAGTATGCC
              |--------------F3----------------------->          |----------------F2-------------------------->

NO          1641
Sequence    ATTAGTGCAA AGAATAGAGC TCGCACCGTA GCTGGTGTCT CTATCTGTAG
                    <--------------------LF-----------------| <--------------------F1c--------------------|

NO          1691
Sequence    TACTATGACC AATAGACAGT TTCATCAAAA ATTATTGAAA TCAATAGCCG
              |----------------------B1c--------------------->              |----------------LB-------------

NO          1741
Sequence    CCACTAGAGG AGCTACTGTA GTAATTGGAA CAAGCAAATT CTATGGTGGT
            ---------> <------------------------B2--------------------|

NO          1791
Sequence    TGGCACAACA TGTTAAAAAC TGTTTATAGT GATGTAGAAA ACCCTCACCT
            <------------------B3-------------------|
```

B)

```
NO          1231
Sequence    GCTCCAGGGC AAACTGGAAA GATTGCTGAT TATAATTATA AATTACCAGA
                       |-----------------F3---------------------->         |-----

NO          1281
Sequence    TGATTTTACA GGCTGCGTTA TAGCTTGGAA TTCTAACAAT CTTGATTCTA
            -------------F2------------------>  <-------------------------LF-----------------------|

NO          1331                                <---------------PNA----------------|
Sequence    AGGTTGGTGG TAATTATAAT TACCGGTATA GATTGTTTAG GAAGTCTAAT
            <-------------------F1c------------------------|          |----------------------B1c------

NO          1381
Sequence    CTCAAACCTT TTGAGAGAGA TATTTCAACT GAAATCTATC AGGCCGGTAG
            ---------------->         |------------------------------LB------------------------->       <-----

NO          1431
Sequence    CACACCTTGT AATGGTGTTG AAGGTTTTAA TTGTTACTTT CCTTTACAAT
             ----------------B2-----------------|                           <------------B3------

NO          1481
Sequence    CATATGGTTT CCAACCCACT AATGGTGTTG GTTACCAACC ATACAGAGTA
            ---------------------------|
```

Red text, L452R (T1355G)
