# Supplementary material for: Detection of SARS-CoV-2 and the L452R spike mutation using reverse transcription loop-mediated isothermal amplification plus bioluminescent assay in real-time (RT-LAMP-BART)
Source: PLoS One. 2022 Mar 21;17(3):e0265748. doi: 10.1371/journal.pone.0265748 (PMC8936440; doi:10.1371/journal.pone.0265748)
Supplement: S4 Fig — Sequence data of amplified products of (A) SARS-RT-LAMP-BART and (B) L452R-RT-LAMP-BART assays. (PDF) [file pone.0265748.s004.pdf]

**Fig. S4. Sequence data of amplified products of (A) SARS-RT-LAMP-BART and (B) L452R-RT-LAMP-BART assays.**

(A) CGTAGCTGGTGTCTCTATCTGTAGTACTATGACCAATAGACAGTTTCATC

(B) AGGTTGGTGGTAATTATAATTACCGGTATAGATTGTTTAGGAAGTCTAATCTCAAACCT

Red text, L452R (T1355G)
