# Supplementary material for: Detection of SARS-CoV-2 and the L452R spike mutation using reverse transcription loop-mediated isothermal amplification plus bioluminescent assay in real-time (RT-LAMP-BART)
Source: PLoS One. 2022 Mar 21;17(3):e0265748. doi: 10.1371/journal.pone.0265748 (PMC8936440; doi:10.1371/journal.pone.0265748)
Supplement: S1 Table — (PDF) [file pone.0265748.s005.pdf]

Table S1. Primer set for real-time RT-PCR [15].

Name	Sequence (5’ to 3’)	Concentration
NIID_2019-nCoV_N_F2	AAA TTT TGG GGA CCA GGA AC	500nM
NIID_2019-nCoV_N_R2ver3	TGG CAC CTG TGT AGG TCA AC	700nM
NIID_2019-nCoV_N_P2	FAM-ATG TCG CGC ATT GGC ATG GA-BHQ	200nM
